# Chrononutrition, Dietary Patterns, and Sleep: A Narrative Review of Current Evidence and Practical Implications

**DOI:** 10.3390/clockssleep8040067

**Published:** 2026-10-15

**Authors:** Andrija Ivanišin, Tamara Sorić, Mladen Mavar, Ivo Klarin, Marijana Matek Sarić

**Affiliations:** 1 Independent Researcher, 10000 Zagreb, Croatia; andrija.ivanisin@gmail.com; 2 Psychiatric Hospital Ugljan, Otočkih Dragovoljaca 42, 23275 Ugljan, Croatia; ravnatelj@pbu.hr; 3 Department of Health Studies, University of Zadar, Splitska 1, 23000 Zadar, Croatia; iklarin@unizd.hr (I.K.); marsaric@unizd.hr (M.M.S.); 4 Department of Gastroenterology, Internal Medicine Clinic, General Hospital Zadar, 23000 Zadar, Croatia

**Keywords:** sleep, nutrition, dietary patterns, chrononutrition, meal timing, circadian rhythms, diet quality, sleep quality

## Abstract

Sleep and nutrition are closely interconnected through behavioral, metabolic, and circadian pathways, yet the strength of evidence varies considerably across dietary exposures. This narrative review synthesizes current evidence on nutrients, foods, dietary patterns, chrononutrition, caffeine and alcohol in relation to sleep, while also considering the effects of insufficient sleep on dietary intake and metabolic health. The relevant literature was identified through searches of PubMed/MEDLINE, Scopus, and Web of Science, supplemented by examination of reference lists and targeted searches. Higher-quality dietary patterns, particularly Mediterranean-style diets, are generally associated with more favorable sleep characteristics, although predominantly observational evidence limits causal interpretation. Intervention studies of individual foods and supplements have reported benefits for selected sleep outcomes, but findings remain heterogeneous and are often based on small samples. Evidence for meal timing and time-restricted eating is similarly inconsistent and does not support a single eating schedule for improving sleep. In contrast, caffeine and alcohol have relatively well-established sleep-disrupting effects. Insufficient sleep can also alter dietary intake, energy balance, glucose regulation, and adiposity, highlighting the bidirectional nature of these relationships. Nutritional approaches may complement broader strategies for sleep and health, but current evidence does not support specific foods, supplements, or eating schedules as stand-alone treatments for sleep problems.

## 1. Introduction

### 1.1. Context and Theoretical Framework

Sleep is a fundamental biological process essential for metabolic homeostasis, cognitive performance, immune function, cardiovascular health, and emotional well-being [1,2]. Increasing evidence indicates that sleep and diet are closely interconnected through hormonal, metabolic, inflammatory, circadian, and gut-microbiota-related pathways, reflecting a bidirectional relationship in which dietary behaviors influence sleep, while sleep characteristics affect food choice, appetite regulation, and energy metabolism [3,4,5]. In this review, the term “diet” is used in a deliberately delimited sense to encompass dietary composition and quality, temporal aspects of eating, and selected dietary exposures relevant to sleep.

Dietary influences on sleep extend beyond individual nutrients to encompass overall dietary patterns and eating behaviors. Greater adherence to healthy dietary patterns, particularly the Mediterranean diet, has generally been associated with more favorable sleep characteristics, whereas diets characterized by high intakes of ultra-processed foods, refined carbohydrates, alcohol, and caffeine have been associated with poorer sleep outcomes [4,6,7]. Conversely, insufficient or disrupted sleep can promote increased energy intake, preference for energy-dense foods, impaired glucose metabolism, and weight gain, reinforcing a cycle that may contribute to obesity and other chronic non-communicable diseases [3,8,9].

Growing interest in chrononutrition has further expanded understanding of the diet–sleep relationship. Beyond dietary composition, the timing, frequency, and regularity of food intake are increasingly recognized as relevant to circadian regulation, metabolic health, and sleep [10,11,12]. Meal timing, time-restricted eating, and alignment of food intake with endogenous circadian rhythms have therefore become important areas of investigation within nutrition and sleep research [13,14,15].

Sleep outcomes considered throughout the review include sleep duration, sleep quality, sleep-onset latency, sleep efficiency, sleep architecture, insomnia symptoms, and daytime sleepiness, as described in greater detail in Section 3.1.

### 1.2. Rationale for the Review and Knowledge Gap

Previous narrative, systematic, and scoping reviews have examined different aspects of the diet–sleep relationship, including individual nutrients and foods, dietary patterns such as the Mediterranean diet, and the temporal dimensions of eating captured within chrononutrition [4,6,7,11,14,16]. Although these reviews have substantially advanced understanding of specific components of the diet–sleep relationship, the evidence remains distributed across several areas of research.

An integrated synthesis that considers dietary composition alongside the temporal dimensions of eating, sleep-disrupting dietary exposures, the metabolic consequences of insufficient sleep, and population-specific considerations is therefore warranted. Such an approach may help place findings concerning individual nutrients, foods, dietary patterns, and meal timing within the broader and bidirectional relationship between nutrition, sleep, and circadian health.

### 1.3. Aim and Scope of the Review

This narrative review aims to integrate current evidence on the relationship between clearly defined nutritional exposures and sleep, with particular emphasis on selected nutrients and foods, overall dietary patterns and diet quality, chrononutrition and meal timing, and sleep-disrupting dietary exposures such as caffeine and alcohol. It also considers the effects of insufficient sleep on dietary intake and metabolic health, as well as nutrition–sleep interactions in populations with distinct physiological, occupational, or clinical characteristics. Particular attention is given to the strength and methodological limitations of the available evidence, areas of uncertainty, and priorities for future research, with the aim of informing evidence-based nutritional recommendations.

The review is guided by three focused questions: (1) how does the evidence for overall dietary patterns compare with that for individual nutrients and foods in relation to sleep outcomes; (2) what is currently known about the contribution of temporal eating patterns and circadian factors to sleep; and (3) to what extent are diet–sleep relationships consistent across populations with different physiological, occupational, or clinical characteristics?

The thematic synthesis follows this framework, progressing from physiological and mechanistic foundations to nutritional exposures, chrononutrition, sleep-disrupting dietary factors, the metabolic consequences of insufficient sleep, and population-specific considerations. A narrative rather than systematic review approach was selected because the aim was to provide an integrated synthesis across a broad and methodologically heterogeneous literature encompassing different dietary exposures, study designs, sleep outcomes, and populations, rather than to address a single narrowly defined intervention or estimate a pooled effect. This approach allows evidence from observational and interventional studies to be integrated with relevant mechanistic and physiological context, while maintaining an appropriate distinction between different types and strengths of evidence.

## 2. Materials and Methods

This narrative review examined the relationship between nutrition, sleep, and circadian health, considering both the potential influence of dietary exposures on sleep and the effects of insufficient or disrupted sleep on dietary intake and metabolic regulation.

### 2.1. Search Strategy

The relevant literature was identified through searches of PubMed/MEDLINE, Scopus, and Web of Science, supplemented by examination of reference lists and targeted searches for specific topics addressed during development of the review. The literature search was conducted without a lower publication date restriction and covered studies available up to 20 August 2026. Further relevant publications were added during manuscript revision in response to reviewers’ comments and to support the revised discussion. Because the purpose was to provide a broad narrative synthesis, searches were topic-driven rather than based on a single predefined systematic search strategy. Representative search combinations included (“sleep” OR “sleep quality” OR “insomnia”) AND (“diet” OR “dietary pattern” OR “nutrition”); (“chrononutrition” OR “meal timing” OR “time-restricted eating”) AND (“circadian rhythm” OR “sleep”); and (“caffeine” OR “alcohol”) AND (“sleep quality” OR “sleep architecture” OR “sleep duration”). Boolean operators (AND/OR), truncation, and, where applicable, Medical Subject Headings (MeSH) terms were used to broaden or refine searches. Additional topic-specific terms were applied as needed for individual nutrients, foods, metabolic outcomes, and population-specific sections, including children and adolescents, pregnancy, older adults, shift workers, and athletes. For dietary patterns and diet quality, topic-specific terms included “dietary pattern”, “diet quality”, “healthy diet”, “Mediterranean diet”, “Dietary Approaches to Stop Hypertension” OR “DASH”, “MIND diet”, “Nordic diet”, “Planetary Health Diet” OR “EAT-Lancet”, “plant-based diet”, “vegetarian diet”, “Western diet”, “Healthy Eating Index”, “dietary inflammatory index”, and “ultra-processed food”.

### 2.2. Inclusion and Exclusion Criteria

Randomized and non-randomized interventions, controlled experimental studies, prospective and cross-sectional observational studies, systematic reviews, and meta-analyses were considered according to their relevance to the questions addressed in the review. Peer-reviewed publications in English were included. Greater emphasis was placed on recent systematic reviews and meta-analyses, controlled studies, and prospective evidence where available, while primary studies were included when they provided important mechanistic or foundational evidence, examined specific foods or populations, or addressed questions for which higher-level evidence was limited. Publications not available in English, non-peer-reviewed publications, and articles that did not address a dietary, nutritional, or temporal eating exposure in relation to sleep or circadian outcomes, or sleep in relation to dietary intake or related metabolic outcomes, were excluded from the synthesis.

Additional exclusion criteria were applied to publications focused primarily on pharmacological sleep treatments, exogenous melatonin, herbal or multi-ingredient supplements, enteral or parenteral nutrition, or critically ill populations, as these fell outside the scope of the review. Studies in which diet or sleep was included only as a covariate rather than as the exposure or outcome of interest were also excluded.

### 2.3. Selection and Synthesis of Evidence

Titles and abstracts identified through the searches were examined for relevance to the scope of the review, and full texts were consulted for publications addressing relationships between dietary or nutritional exposures and sleep, circadian rhythms, or related metabolic outcomes. Reference lists of relevant reviews and included articles were also examined to identify additional publications. A.I., T.S., and M.M.S. contributed to literature identification and selection.

The individual nutrients and foods discussed in greater detail were selected as illustrative examples based on the availability of human intervention evidence and, where available, supporting evidence syntheses. Selection also considered their relevance to distinct biological pathways implicated in sleep regulation. For foods, preference was given to whole foods or commonly consumed dietary components rather than isolated extracts or pharmacological preparations.

Evidence was synthesized narratively. When available and relevant, sample sizes, estimates of between-group differences or associations, confidence intervals (CIs), and reported *p*-values were extracted for descriptive reporting; no effect sizes were calculated or pooled specifically for this review. Interpretation considered study design, population characteristics, methods used to assess diet and sleep, potential confounding, and consistency of findings across studies. Particular attention was given to temporality and reverse causation because the relationship between nutrition and sleep is potentially bidirectional. Observational associations were therefore interpreted separately from findings obtained through experimental sleep manipulation or dietary interventions. Consistent with the narrative design of the review, a predefined systematic review protocol was not developed, the Preferred Reporting Items for Systematic Reviews and Meta-Analyses (PRISMA) reporting guidelines were not applied, independent duplicate screening was not performed, and no formal risk-of-bias assessment or certainty-of-evidence grading was conducted.

To strengthen methodological transparency and reporting, the manuscript was evaluated with reference to the Scale for the Assessment of Narrative Review Articles (SANRA) [17]. The assessment considered the six SANRA domains: justification of the review’s importance, statement of aims, description of the literature search, referencing, scientific reasoning, and appropriate presentation of relevant outcome data. A descriptive overview of how these criteria were addressed is provided in Table S1. SANRA was applied as a framework for evaluating the narrative review itself rather than as a risk-of-bias assessment of the individual studies included in the synthesis.

## 3. Thematic Synthesis of Evidence

### 3.1. Physiological and Mechanistic Basis

Human sleep is organized into non-rapid eye movement (NREM) and rapid eye movement (REM) sleep, which alternate cyclically across the night. Sleep timing and architecture are regulated primarily through the interaction between the homeostatic sleep drive, which increases with time spent awake, and the circadian timing system [18,19]. The suprachiasmatic nucleus of the hypothalamus acts as the central circadian pacemaker and synchronizes endogenous rhythms primarily to the light–dark cycle, while neural circuits involving the hypothalamus and brainstem regulate transitions between wakefulness, NREM sleep, and REM sleep [18,19]. Melatonin secretion increases in the evening under dim-light conditions and provides an endogenous signal of circadian night, whereas cortisol follows a circadian rhythm characterized by low concentrations during the early part of the night and a rise toward the morning [20,21]. Disruption of circadian alignment, as occurs during night-shift work or recurrent changes in sleep–wake timing, is associated with adverse sleep and metabolic outcomes [22].

For clarity, this review considers several distinct sleep domains that should not be regarded as interchangeable. Sleep duration refers to the total time spent asleep, whereas sleep quality is a broader and predominantly subjective construct reflecting an individual’s perception of sleep and is commonly assessed using validated questionnaires such as the Pittsburgh Sleep Quality Index (PSQI) [23]. Sleep-onset latency refers to the time required to fall asleep, and sleep efficiency to the proportion of time in bed spent asleep. Both can be estimated subjectively using sleep diaries or assessed objectively using polysomnography or actigraphy [24,25]. Sleep architecture describes the organization and distribution of NREM and REM sleep stages across the night and is typically assessed using polysomnography [25]. Insomnia symptoms are commonly evaluated using validated self-report instruments such as the Insomnia Severity Index [26], whereas daytime sleepiness is most often assessed subjectively, for example using the Epworth Sleepiness Scale [27], although objective methods such as the Multiple Sleep Latency Test are also available [28]. Subjective and objective measures capture partly different aspects of sleep and do not necessarily yield concordant findings. Accordingly, where reported in the primary studies, this review distinguishes between subjectively and objectively assessed sleep outcomes.

Chronotype reflects interindividual differences in the preferred timing of sleep and daily activity and is influenced by biological, environmental, and behavioral factors [13,29]. It can be assessed using instruments such as the Morningness–Eveningness Questionnaire (MEQ) [30] and Munich Chronotype Questionnaire (MCTQ) [31]. Later chronotypes tend to have later sleep and meal timing and may experience shorter sleep duration and greater social jetlag when social schedules require early waking [29]. A systematic review also found that evening chronotype is associated with less favorable eating behaviors, including later eating, more frequent breakfast skipping, and greater consumption of processed or ultra-processed foods, although the predominantly observational nature of the evidence precludes causal interpretation [32].

Homeostatic sleep regulation also provides a direct mechanistic link between sleep physiology and dietary exposures. Extracellular adenosine accumulates during prolonged wakefulness and contributes to increasing sleep pressure, while caffeine, one of the most widely consumed dietary stimulants, promotes wakefulness primarily through antagonism of adenosine receptors [33,34]. The effects of caffeine on sleep outcomes, dose and timing, habitual consumption, and interindividual variability are discussed in Section 3.2.4.

Sleep and circadian regulation are closely integrated with metabolic function. Experimental sleep restriction and circadian misalignment have been shown to impair glucose tolerance and insulin sensitivity, while the timing of food intake relative to endogenous circadian rhythms can influence metabolic responses [10,35]. These interactions provide a physiological basis for chrononutrition, in which the timing of energy and nutrient intake is considered alongside dietary composition in relation to circadian and metabolic regulation [10,13].

The gut microbiota represents another potential pathway linking diet and sleep, although the evidence is less established than that for circadian and metabolic mechanisms. Gut microorganisms influence tryptophan metabolism and produce metabolites, including short-chain fatty acids, bile acids, and tryptophan-derived compounds, that participate in neural, endocrine, immune, and metabolic signaling along the microbiota–gut–brain axis [5,36]. Most peripheral serotonin is synthesized by enterochromaffin cells in the gastrointestinal tract, and gut microorganisms can modulate intestinal serotonin production and tryptophan metabolism. Importantly, peripheral serotonin does not cross the blood–brain barrier [5]. Experimental evidence in mice indicates that substantial manipulation of the gut microbiota can alter sleep–wake architecture and sleep electroencephalographic characteristics [37]. Evidence linking the gut microbiota and microbial metabolites with sleep is growing, but much of the mechanistic evidence remains preclinical and causal pathways in humans are not yet well-established [36]. The microbiota–gut–brain axis should therefore be regarded as a plausible and emerging component of the diet–sleep relationship rather than an established mechanism through which specific foods or dietary patterns improve sleep.

### 3.2. Nutritional and Dietary Factors in the Bidirectional Relationship with Sleep

Table 1 provides a descriptive overview of representative primary studies and evidence syntheses across the nutritional, dietary, and sleep-related exposures discussed below. Because these publications differ in design, population, and outcome measurement, the results are presented without assigning common evidence grades or implying that their effect estimates are directly comparable.

#### 3.2.1. Nutrients Relevant to Sleep Regulation

Against this physiological background, several nutrients have been investigated in relation to sleep because of their roles in neurotransmitter synthesis, neuronal function, circadian signaling, and metabolic regulation. Tryptophan, omega-3 fatty acids, magnesium, and vitamin D were selected as illustrative examples because human studies, including intervention studies and evidence syntheses, have investigated their relationships with sleep and because they represent distinct biological pathways. This selection is not exhaustive. Their physiological relevance does not, by itself, establish that supplementation improves sleep.

Tryptophan has received particular attention because it is an essential amino acid and a precursor of serotonin and melatonin, both involved in sleep–wake regulation [61]. Its availability to the brain depends not only on absolute intake but also on competition with other large neutral amino acids for transport across the blood–brain barrier [62,63]. In a systematic review of 18 studies, Sutanto et al. [38] found that tryptophan supplementation was associated with shorter wake after sleep onset (WASO), while no significant effects were observed for other assessed sleep components. Participants receiving ≥1 g/day had shorter WASO than those receiving <1 g/day (28.91 vs. 56.55 min, respectively; *p* = 0.001) [38]. These findings suggest a possible dose-related effect on sleep continuity, but do not establish a general benefit of tryptophan supplementation across sleep outcomes.

Long-chain omega-3 polyunsaturated fatty acids have also been investigated in relation to sleep. Omega-3 fatty acids have biological roles in neuronal membrane function and have been proposed to influence sleep and circadian processes through several pathways, including serotonergic and melatonergic signaling; however, the mechanisms underlying their effects on human sleep remain incompletely understood [64,65]. A 2024 systematic review and meta-analysis of eight randomized controlled trials found that omega-3 fatty acid supplementation modestly increased sleep efficiency compared with control conditions (mean difference: 1.88 percentage points; 95% CI: 1.00–2.77 percentage points), while no significant effects were observed for sleep latency or total sleep duration. Subjectively assessed sleep also improved, although heterogeneity across studies was high (I^2^ = 88%), warranting cautious interpretation [39]. The available evidence therefore points to possible benefits for selected sleep outcomes rather than a consistent effect across all dimensions of sleep.

Magnesium has been studied in relation to sleep because of its involvement in neuronal and neuroendocrine processes, including the regulation of neuronal excitability and neurotransmitter function [66]. Observational studies generally suggest associations between magnesium status and sleep outcomes, but intervention findings are less consistent [67]. In a systematic review and meta-analysis of three randomized controlled trials involving 151 older adults with insomnia, magnesium supplementation reduced sleep-onset latency by 17.36 min compared with placebo (95% CI: −27.27 to −7.44), whereas the approximately 16-min increase in total sleep time was not statistically significant [40]. All three trials were judged to have a moderate-to-high risk of bias, and the certainty of evidence was low to very low [40]. Baseline magnesium status may influence intervention findings, but it remains difficult to characterize accurately [66].

Vitamin D status has repeatedly been associated with sleep outcomes. A meta-analysis of nine observational studies involving 9397 participants found that vitamin D deficiency was associated with 50% higher odds of sleep disorders [odds ratio (OR) 1.50, 95% CI: 1.31–1.72], as well as higher odds of poor sleep quality (OR 1.59, 95% CI: 1.23–2.05) and short sleep duration (OR 1.74, 95% CI: 1.30–2.32) [41]. Because most included studies were cross-sectional, these associations cannot establish directionality or causality. Intervention evidence is less extensive. A subsequent systematic review and meta-analysis found that vitamin D supplementation improved PSQI scores compared with placebo, but evidence for effects on sleep duration and specific sleep disorders remained inconclusive [68]. Interpretation of supplementation studies should account for baseline vitamin D status and the considerable interindividual variation in vitamin D requirements and response to supplementation [69,70]. Differences in baseline status, population characteristics, supplementation regimens, and sleep assessment methods may partly explain variation between studies.

Findings for the selected nutrients vary depending on the sleep outcome, study population, baseline nutritional status, dose, and study design. Most intervention studies have also examined supplementation rather than habitual dietary intake.

#### 3.2.2. Foods, Dietary Patterns, and Sleep Quality

Evidence on diet and sleep extends beyond individual nutrients to the overall quality and composition of habitual diets. A systematic review of 29 studies found that consumption of healthier foods and dietary patterns was generally associated with better sleep quality, whereas greater consumption of processed and free-sugar-rich foods was associated with less favorable sleep characteristics [4]. The predominantly observational nature of this literature remains an important limitation, as poor or insufficient sleep can itself influence appetite, food preferences, and dietary intake [59,71].

Among specific dietary patterns, the Mediterranean diet has received particular attention. A systematic review of 23 reports found that greater adherence to the Mediterranean diet was generally associated with better sleep quality and a lower likelihood of inadequate sleep duration, excessive daytime sleepiness, and insomnia symptoms [7]. A separate systematic review and meta-analysis examining dietary patterns and insomnia reported lower odds of insomnia symptoms among individuals with greater adherence to the Mediterranean diet (OR 0.86, 95% CI: 0.79–0.93) [42]. These findings are consistent with a favorable relationship between Mediterranean diet adherence and sleep, but most of the available studies are observational. Differences in dietary assessment, sleep measures, study populations, and adjustment for confounding factors further limit causal interpretation.

The association between Mediterranean diet adherence and sleep is unlikely to reflect the action of a single nutrient or food. The Mediterranean diet is characterized by high consumption of vegetables, fruits, legumes, nuts, whole grains, and olive oil, together with regular consumption of fish, and provides fiber, unsaturated fatty acids, polyphenols, and a range of micronutrients [72]. The combined nutritional profile of this dietary pattern may contribute to the observed associations with sleep, although the underlying mechanisms remain incompletely understood [73]. Mediterranean diet adherence may also cluster with other health-related behaviors, including greater physical activity and lower smoking prevalence [74]. Residual confounding by lifestyle, socioeconomic factors, body weight, and underlying health therefore remains difficult to exclude [7].

Beyond the Mediterranean diet, other healthy dietary patterns and diet-quality indices have also been examined in relation to sleep, although the evidence base is smaller and predominantly observational. For the Dietary Approaches to Stop Hypertension (DASH) diet, a cross-sectional study of 535 Iranian adults found that higher adherence was associated with lower odds of poor sleep quality and short sleep duration, particularly among women. In that study, both sleep quality and duration were assessed subjectively using the PSQI [75]. In another cross-sectional study of 337 Iranian employees, in which sleep was also assessed using the PSQI, higher DASH adherence was associated with lower odds of short sleep duration (OR 0.53, 95% CI: 0.30–0.96) but not with poor sleep quality (OR 0.69, 95% CI: 0.38–1.27) [76]. With respect to dietary patterns that also consider environmental sustainability, a cross-sectional analysis of 9041 Chinese adults aged ≥65 years found that higher adherence to the Planetary Health Diet, assessed using the Planetary Health Diet Index, was associated with higher odds of good self-reported sleep quality (OR 1.35, 95% CI: 1.20–1.52) and adequate self-reported sleep duration (OR 1.36, 95% CI: 1.20–1.54), both obtained through face-to-face interviews [77]. Prospective evidence has also emerged from the UK Biobank, where greater adherence to the Planetary Health Diet was associated with a lower risk of incident sleep disorders identified from linked health records during long-term follow-up [78]. At the level of overall diet quality, a meta-analysis of five observational studies found that higher Healthy Eating Index scores were associated with lower odds of poor sleep quality and abnormal sleep duration. The included studies assessed sleep using subjective measures, including the PSQI, the Sleep Disorders Questionnaire, and self-reported sleep duration [79]. A mini-review of plant-rich diets, including the DASH, MIND (Mediterranean–DASH Intervention for Neurodegenerative Delay), and vegetarian diets, concluded that these patterns may be favorably associated with sleep, while emphasizing that the available evidence remains limited [80]. These findings suggest that favorable sleep characteristics may be associated with broader features shared by healthy, predominantly plant-based dietary patterns—higher intakes of vegetables, fruits, legumes, whole grains, and nuts, and lower intakes of processed foods and added sugars—rather than with characteristics unique to a single dietary pattern. However, the evidence for dietary patterns other than the Mediterranean diet remains comparatively limited and predominantly observational, with substantial heterogeneity in populations, dietary-pattern definitions, sleep outcomes, and methods of sleep assessment.

Less favorable dietary patterns have also been examined. A systematic review and meta-analysis of 15 cross-sectional studies found that higher consumption of ultra-processed foods was associated with adverse sleep-related outcomes across children, adolescents, and adults [81]. A subsequent meta-analysis found that higher ultra-processed food consumption was associated with greater odds of insomnia (OR 1.53, 95% CI: 1.20–1.95), although subgroup analyses indicated that this association was significant in adolescents but not in adults [43]. These findings strengthen the epidemiological evidence for a relationship between ultra-processed food consumption and sleep, but do not establish that food processing itself causes sleep disturbance. Reverse causality also remains possible, further limiting causal interpretation of these associations.

Among individual foods, the following examples were selected because human intervention evidence is available, although the evidence base is smaller and less consistent than that for broader dietary patterns. In an uncontrolled intervention involving 24 adults with self-reported sleep problems, consumption of two kiwifruits one hour before bedtime for four weeks was associated with improvements in several sleep outcomes assessed using the PSQI, sleep diaries, and actigraphy [44]. However, the absence of a parallel control group and the small sample substantially limit causal interpretation and generalizability. Evidence syntheses have also identified potentially favorable effects of melatonin-containing foods, including kiwifruit, on sleep outcomes, although substantial heterogeneity in study populations, intervention protocols, and sleep assessment methods limits firm conclusions [82].

Tart cherry products have also been investigated, partly because tart cherries contain melatonin and polyphenolic compounds. In a randomized, double-blind, placebo-controlled crossover pilot trial of adults aged >50 years with insomnia, 11 participants were randomized and eight were included in the analysis. Tart cherry juice increased polysomnographic sleep time by 84 min compared with placebo, while polysomnographic sleep efficiency did not improve significantly [45]. However, the very small sample limits the interpretation of these findings. More recent intervention evidence has been inconsistent. A randomized placebo-controlled crossover trial of Montmorency tart cherry powder involving 34 adults with overweight or obesity found no significant differences in objectively or subjectively assessed sleep outcomes [46]. Differences in study populations, formulations, doses, and outcome assessment make direct comparison difficult, and the current evidence is insufficient to support tart cherry products as an established intervention for insomnia.

Milk and dairy products have also been examined in relation to sleep. A systematic review of 14 observational and intervention studies reported potentially favorable associations or effects of milk and dairy consumption on sleep, although findings varied considerably across populations, dairy products, and study designs [83]. A meta-analysis of clinical trials found improvement in self-reported sleep quality assessed using the PSQI, but only a small number of studies contributed to the quantitative analysis [47]. The heterogeneity of dairy products and intervention protocols currently precludes specific recommendations regarding the type, amount, or timing of dairy consumption for improving sleep.

Fatty fish provides another example of a whole-food intervention in which several potentially relevant nutrients are consumed simultaneously. In a randomized trial involving 95 male forensic inpatients, consumption of Atlantic salmon three times per week for approximately five months prevented the increase in actigraphy-derived sleep latency observed in the meat-based control group and was associated with better self-reported daily functioning at post-intervention [48]. Vitamin D status was positively associated with actigraphy-derived sleep efficiency at baseline and self-reported sleep quality at post-intervention, suggesting that factors in addition to omega-3 fatty acids may have contributed to the observed associations, although these findings do not establish the relative contribution of individual nutrients [48]. The specific study population and limited replication further restrict the generalizability of these findings.

**Figure 1:**
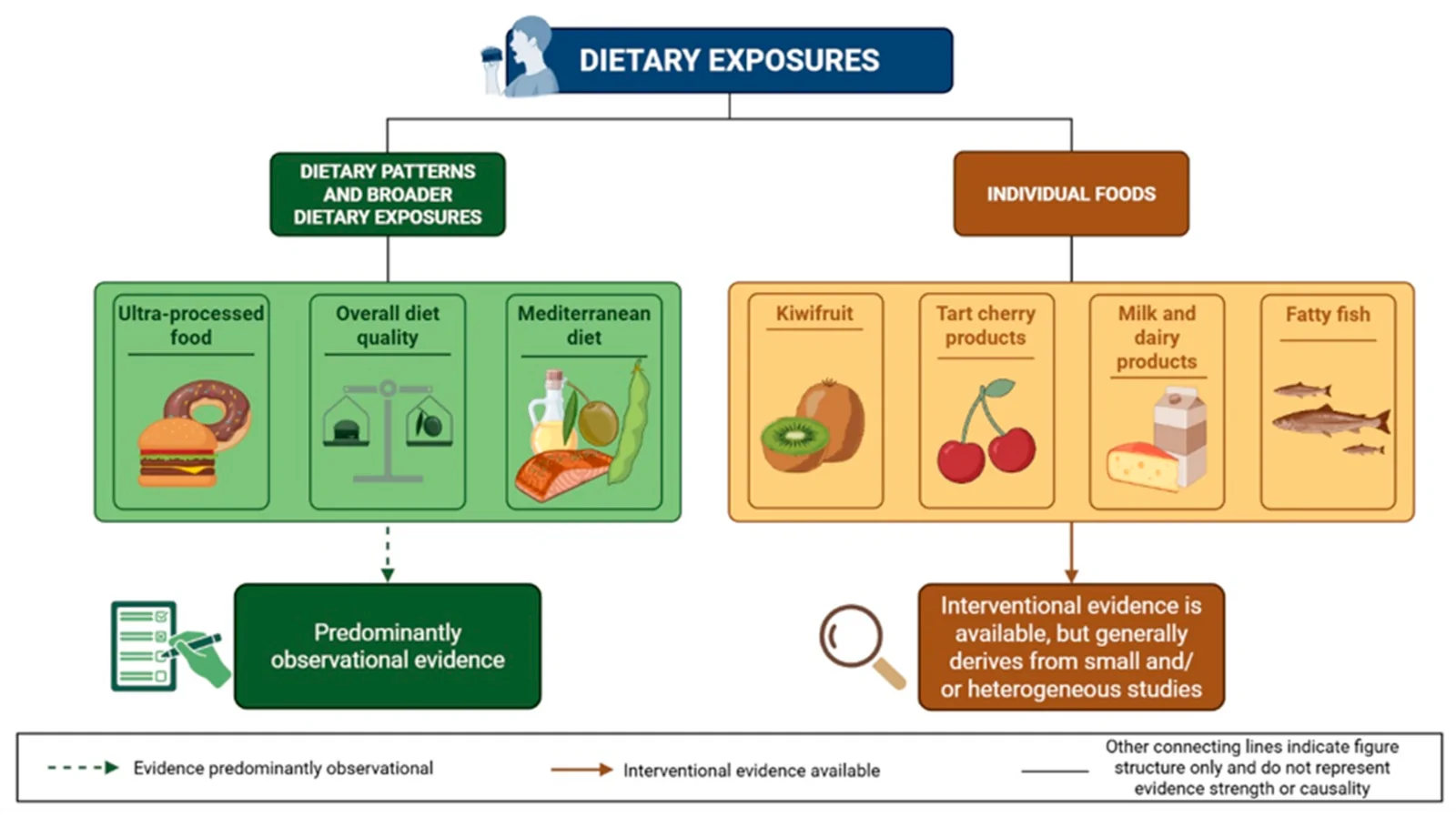
Structure and broad evidence characteristics of dietary exposures examined in relation to sleep. Dietary exposures are grouped into dietary patterns and broader dietary exposures, including ultra-processed food consumption, overall diet quality, and Mediterranean-style dietary patterns, and selected individual foods, including kiwifruit, tart cherry products, milk and dairy products, and fatty fish. Evidence concerning broader dietary patterns and exposures is predominantly observational, whereas interventional evidence is available for the selected individual foods, although these studies are generally small and/or heterogeneous in design, population, and sleep assessment. Dashed and solid arrows distinguish predominantly observational evidence from exposure groups for which interventional evidence is available, while other connecting lines indicate figure structure only. The grouping is descriptive and should not be interpreted as a formal grading of evidence certainty. Created in BioRender. Sorić, T. (2026) https://BioRender.com/nkvrvfz.

The main characteristics of the evidence discussed in this section are summarized in Figure 1. Overall, associations between diet quality and sleep are supported more consistently than sleep-promoting effects of individual foods, for which intervention evidence remains limited and heterogeneous. Dietary composition, however, represents only one dimension of nutritional exposure; the timing and regularity of food intake in relation to endogenous circadian rhythms may also influence sleep and metabolic regulation, providing the basis for chrononutrition.

#### 3.2.3. Chrononutrition, Meal Timing, and Sleep

Chrononutrition encompasses several temporal dimensions of eating, including meal timing, the distribution of energy intake across the day, eating regularity and frequency, and the duration of the daily eating window [11,13]. These dimensions are not interchangeable, and their definition and measurement vary considerably across studies [84]. The principal chrononutrition dimensions relevant to sleep and their assessment in human studies are summarized in Table 2.

Later eating is among the most frequently studied temporal dietary exposures in relation to sleep. In a systematic scoping review, Saidi et al. [11] identified 35 studies examining meal timing, including 16 addressing late eating and six comparing earlier and later meal schedules. The findings provided preliminary support for relationships between the temporal characteristics of eating and several dimensions of sleep, but substantial heterogeneity in study design, populations, and exposure and outcome assessment precluded quantitative synthesis.

Recent population-based studies provide further evidence of an association between later eating and poorer sleep. In an analysis of 7023 US adults, later timing of the first meal, eating midpoint, and last meal was associated with poorer self-reported sleep quality, reflected in higher PSQI scores after adjustment for demographic and lifestyle factors [49]. Compared with the earliest tertile, the latest tertile was associated with PSQI scores higher by 0.55 points for first-meal timing, 0.59 points for eating midpoint, and 0.39 points for last-meal timing [49]. In 379 midlife Mexican women, later meal timing and a shorter interval between the last meal and bedtime were associated with longer self-reported sleep latency and poorer self-reported sleep quality, while associations with sleep duration varied according to the temporal exposure examined [85]. These findings suggest that both clock time and the timing of eating relative to sleep may be relevant, but their observational nature does not establish directionality.

Experimental studies have produced less consistent findings. In a randomized crossover study of 20 healthy adults, consuming dinner one hour rather than five hours before bedtime did not significantly alter polysomnographically assessed sleep-stage distribution, although electroencephalographic spectral analysis showed an initial 2.5% increase in delta power and a 2.7% decrease in combined alpha and beta power [50]. The same experimental protocol showed marked effects of late dinner on nocturnal glucose and lipid metabolism despite the absence of major changes in sleep architecture [86]. This distinction suggests that metabolic consequences of late eating should not be assumed to correspond directly to measurable deterioration in sleep.

Chronotype may complicate the interpretation of associations between meal timing and sleep, as meal timing and sleep timing are closely correlated behaviors. For example, a study of 162 university students found poorer self-reported sleep quality, assessed using the PSQI, among later sleepers, but no independent association between main- or last-meal timing and sleep quality [87]. These findings highlight the importance of accounting for chronotype and sleep timing rather than interpreting clock-based meal timing in isolation.

Time-restricted eating (TRE), in which daily food intake is confined to a defined eating window, has received increasing attention as a chrononutrition intervention. Evidence for its effects on sleep remains mixed. A systematic review of randomized controlled trials found that short- to medium-term TRE generally did not worsen sleep, but improvements were inconsistent across studies and sleep outcomes [88]. Another systematic review similarly concluded that most studies did not demonstrate significant changes in sleep, while also highlighting substantial variation in TRE protocols and sleep assessment methods [89].

Results from individual TRE trials also vary. In adults with obesity, 12 months of 8-h TRE did not significantly change self-reported sleep quality, sleep duration, insomnia severity, or obstructive sleep apnea risk compared with daily calorie restriction or control conditions [90]. Similarly, a randomized trial comparing early, late, and self-selected 8-h TRE schedules in adults with overweight or obesity found no significant between-group differences in objectively assessed sleep outcomes after 12 weeks [91]. In contrast, a post hoc analysis of a 12-week randomized isocaloric feeding study in adults with obesity and prediabetes or diabetes found that a 10-h early TRE schedule increased actigraphy-derived total sleep time by 55 min and shifted sleep midpoint 44 min earlier compared with a usual eating pattern, although sleep continuity did not differ between groups [51]. In a randomized trial of 30 young, normal-weight adults with insufficient or poor sleep, an 8-week self-selected 10-h TRE intervention resulted in actigraphy-derived earlier sleep onset, later wake time, and fewer movements during sleep compared with controls, although sleep duration did not differ between groups [52]. Recent meta-analytic evidence found small improvements in sleep duration and PSQI scores when changes within TRE groups were pooled; however, these effects were not significant in analyses restricted to controlled trials [92].

Current evidence does not demonstrate a consistent effect of TRE on sleep. Differences in eating-window duration and timing, baseline sleep status, population characteristics, intervention duration, and sleep assessment methods may partly explain the variation between studies. Interpretation is further complicated by differences in habitual sleep schedules and chronotype, which can alter the biological timing of a given eating window. Many trials were also designed primarily to investigate body weight or cardiometabolic outcomes, with sleep assessed as a secondary endpoint [88,89].

#### 3.2.4. Caffeine, Alcohol, and Other Dietary Exposures Relevant to Sleep

Caffeine is among the most extensively studied dietary factors affecting sleep. A systematic review and meta-analysis of 24 studies found that caffeine reduced total sleep time by approximately 45 min and sleep efficiency by 7 percentage points, while increasing sleep-onset latency by 9 min and WASO by 12 min. Caffeine also altered sleep architecture, increasing light sleep by approximately 6 min and reducing deep sleep by approximately 11 min [93]. Considerable interindividual variability in caffeine metabolism and sensitivity has been documented and is influenced by genetic and non-genetic factors, including variation in cytochrome P450 1A2 (CYP1A2), the principal enzyme responsible for caffeine metabolism [94,95]. The extent to which *CYP1A2* genotype alone can inform individualized caffeine-timing recommendations remains insufficiently established. Experimental evidence also indicates that caffeine effects depend on the amount consumed and its timing. In a double-blind randomized crossover trial of 23 men, 100 mg of caffeine consumed 4, 8, or 12 h before bedtime did not significantly alter objective or subjective sleep compared with placebo. In contrast, 400 mg delayed sleep initiation and altered sleep architecture even when consumed 12 h before bedtime, with greater disruption when intake occurred closer to bedtime. When consumed 4 h before bedtime, 400 mg also reduced subjective sleep quality by 34% [53]. Consistent with these findings, a meta-analysis of controlled crossover trials found reductions in total sleep time (−34.67 min) and sleep efficiency (−4.74 percentage points), together with an 8.35-min increase in sleep-onset latency following caffeine consumption [54].

Regular caffeine consumption does not necessarily remove these effects. Weibel et al. [96] studied 20 healthy men who habitually consumed approximately 478 mg caffeine/day. During the caffeine condition, participants received 150 mg three times daily for 10 days. Total sleep time and overall sleep architecture did not differ significantly from placebo, but REM sleep latency was longer and the accumulation of REM sleep was delayed. Participants also reported greater difficulty waking and more tiredness after waking [96].

Alcohol affects sleep differently. A recent systematic review and meta-analysis of 27 studies found that alcohol delayed the onset of REM sleep and reduced REM sleep duration [55]. REM disruption was already apparent at doses ≤ 0.50 g/kg and became greater with increasing doses. A dose of 0.50 g/kg corresponds to approximately 35 g of alcohol in a 70 kg adult, or about 2.5 US standard drinks when one standard drink is defined as 14 g of pure alcohol [97]. This equivalence varies according to body weight and the amount of pure alcohol used to define a standard drink, which differs across countries [98]. By contrast, shorter sleep-onset latency was observed only at doses ≥ 0.85 g/kg, corresponding to approximately 60 g of alcohol in a 70 kg adult, or about 4.3 US standard drinks under the same assumption. Effects on total sleep time, sleep efficiency, and WASO were less certain. Thus, at higher doses, alcohol may shorten sleep latency, but this occurs alongside greater disruption of REM sleep and does not indicate better overall sleep [55].

This pattern has also been examined during repeated exposure. McCullar et al. [56] studied 30 adults during three consecutive nights of pre-sleep alcohol or placebo in a crossover design. Alcohol consumption ended one hour before bedtime and was associated with greater slow-wave sleep during the first part of the night, delayed REM sleep onset, and reduced REM sleep. These alterations in sleep architecture remained evident across three consecutive nights of pre-sleep alcohol consumption [56]. Longitudinal evidence also links alcohol consumption with subsequent sleep problems. A meta-analysis of 11 cohort studies found higher odds of incident sleep disorders among drinkers (OR 1.37, 95% CI: 1.22–1.54), although the association was not significant in the analysis restricted to heavy drinking and heterogeneity in that analysis was high [57].

Energy drinks are also relevant because they typically contain high amounts of caffeine and are commonly consumed to maintain wakefulness or alertness, particularly during studying and examinations [99]. In a cross-sectional national study of 53,266 Norwegian college and university students aged 18–35 years, increasing frequency of energy-drink consumption was associated with progressively shorter sleep duration, lower sleep efficiency, longer sleep-onset latency, and more wake time after sleep onset [58]. Daily consumption was associated with approximately twice the risk of sleeping <6 h compared with seldom or no consumption (risk ratio 2.07 in men and 1.87 in women). The association was evident even at lower frequencies of consumption, although the cross-sectional design does not establish whether energy drinks contribute to sleep loss or are consumed in response to insufficient sleep [58].

The role of sugar is less clear. A systematic review and meta-analysis of observational studies found that short sleep duration was associated with higher sugar-sweetened beverage consumption, but findings for total sugar intake were less consistent and differed between children and adults [100]. Importantly, these studies generally examined short sleep as the exposure and sugar or sugar-sweetened beverage intake as the outcome, rather than demonstrating that sugar intake subsequently disrupts sleep. These findings therefore provide limited evidence that sugar itself acts as an independent sleep-disrupting exposure.

These findings also illustrate the bidirectional nature of the diet–sleep relationship, as some dietary exposures can influence sleep, while insufficient sleep may in turn alter subsequent dietary behavior.

#### 3.2.5. Consequences of Insufficient Sleep for Dietary Intake and Metabolic Health

Experimental studies provide evidence that restricting sleep can alter subsequent energy intake and eating behavior. In a meta-analysis of 41 randomized controlled trials, sleep restriction increased subjective hunger by 13.4 mm on a 100-mm visual analogue scale, in which higher scores indicate greater perceived hunger, and energy intake by approximately 253 kcal/day compared with the non-restricted control sleep condition. Also, partial sleep restriction was associated with a small increase in body weight of 0.34 kg [59]. A subsequent systematic review of 50 randomized controlled trials also reported increases in eating occasions, portion size, and fat intake during sleep restriction, although findings were not uniform across studies [71].

Changes in food intake following sleep restriction appear to involve more than physiological hunger. In a crossover study of 12 healthy young men, two nights of restricted sleep increased hunger by 24% and appetite by 23%, with the largest increases reported for energy-dense foods with high carbohydrate content [101]. Sleep restriction has also been associated with greater neural responses to food stimuli, while longer waking periods may additionally increase opportunities for eating [102]. These pathways are difficult to separate under free-living conditions and may not contribute equally across individuals.

Changes in leptin and ghrelin are often used to explain the relationship between insufficient sleep and increased food intake, but this model is less consistent than is sometimes suggested. Studies have reported changes in both hormones following sleep restriction, yet the direction and magnitude of these responses vary according to study design, participant characteristics, energy balance, and duration of sleep restriction. A systematic review of clinical trials found no consistent effect of sleep duration on circulating leptin or ghrelin despite observed changes in appetite and dietary intake [71]. Increased food intake following insufficient sleep should therefore not be assumed to result from a uniform pattern of reduced leptin and increased ghrelin.

The metabolic effects introduced in Section 3.1 have been examined further in controlled sleep-manipulation studies. In a systematic review and meta-analysis of randomized controlled trials, Sondrup et al. [103] found that sleep restriction reduced insulin sensitivity assessed using oral or intravenous glucose tolerance tests and homeostatic model assessment of insulin resistance. Whole-body insulin sensitivity measured using the hyperinsulinemic–euglycemic clamp was also reduced after short sleep, whereas peripheral insulin sensitivity was not significantly affected. Circadian misalignment and slow-wave sleep suppression were likewise associated with reduced insulin sensitivity [103].

Changes in energy balance may also be accompanied by changes in fat distribution. Covassin et al. [104] studied 12 healthy, non-obese adults during two 21-day inpatient sessions that included 14 days of either restricted or control sleep opportunities with unrestricted access to food. Sleep restriction increased energy intake without a corresponding increase in energy expenditure and resulted in weight gain. Abdominal total fat increased by 9% and visceral abdominal fat by 11% [104]. Although the sample was small and the experimental conditions do not reproduce habitual sleep loss over longer periods, these findings suggest that changes in body weight alone may not capture the metabolic consequences of prolonged sleep restriction.

Tasali et al. [60] randomized 80 adults with overweight who habitually slept less than 6.5 h/night to individualized sleep-hygiene counseling aimed at extending sleep or to continuation of habitual sleep. Participants were instructed to maintain their usual diet and physical activity. Sleep duration increased by approximately 1.2 h/night in the intervention group relative to controls, while objectively assessed energy intake decreased by 270 kcal/day. Because the intervention targeted sleep rather than diet, the study provides particularly relevant evidence that increasing sleep duration in habitual short sleepers can influence energy intake under free-living conditions.

Increasing sleep duration refers specifically to correcting habitually insufficient sleep rather than prolonging sleep beyond recommended levels. Current recommendations indicate that healthy adults should generally obtain at least 7 h of sleep per night [105], with 7–9 h recommended for adults aged 18–64 years and 7–8 h for adults aged ≥65 years [106]. Habitually long sleep should not be interpreted as a desirable target. A systematic review and meta-analysis found that long sleep duration was associated with higher risks of mortality, diabetes, cardiovascular disease, stroke, and obesity [107]. These associations are observational and may partly reflect underlying illness, behavioral factors, or reverse causation. The findings of Tasali et al. [60] should therefore be interpreted as evidence concerning the correction of insufficient sleep rather than as evidence that progressively longer sleep is beneficial.

Whether these effects translate into long-term changes in body weight is less certain. In an updated meta-analysis, Bacaro et al. [108] included 12 prospective studies comprising 154,936 adults and found that short sleep was associated with a higher risk of developing obesity (OR 1.41, 95% CI: 1.18–1.69). Long sleep was not significantly associated with incident obesity. Although prospective designs strengthen temporality, residual confounding by lifestyle, psychosocial, and socioeconomic factors remains difficult to exclude [108].

The relationship can also operate in the opposite direction. Obesity is an important modifiable risk factor for obstructive sleep apnea (OSA), although upper-airway anatomy, age, sex, and other factors also contribute to OSA risk [109,110]. Sleep fragmentation and intermittent hypoxia associated with OSA can, in turn, contribute to impaired glucose regulation and other aspects of metabolic dysfunction [111,112]. These reciprocal associations suggest that increasing adiposity may reinforce, rather than simply result from, adverse sleep–metabolic interactions.

**Figure 2:**
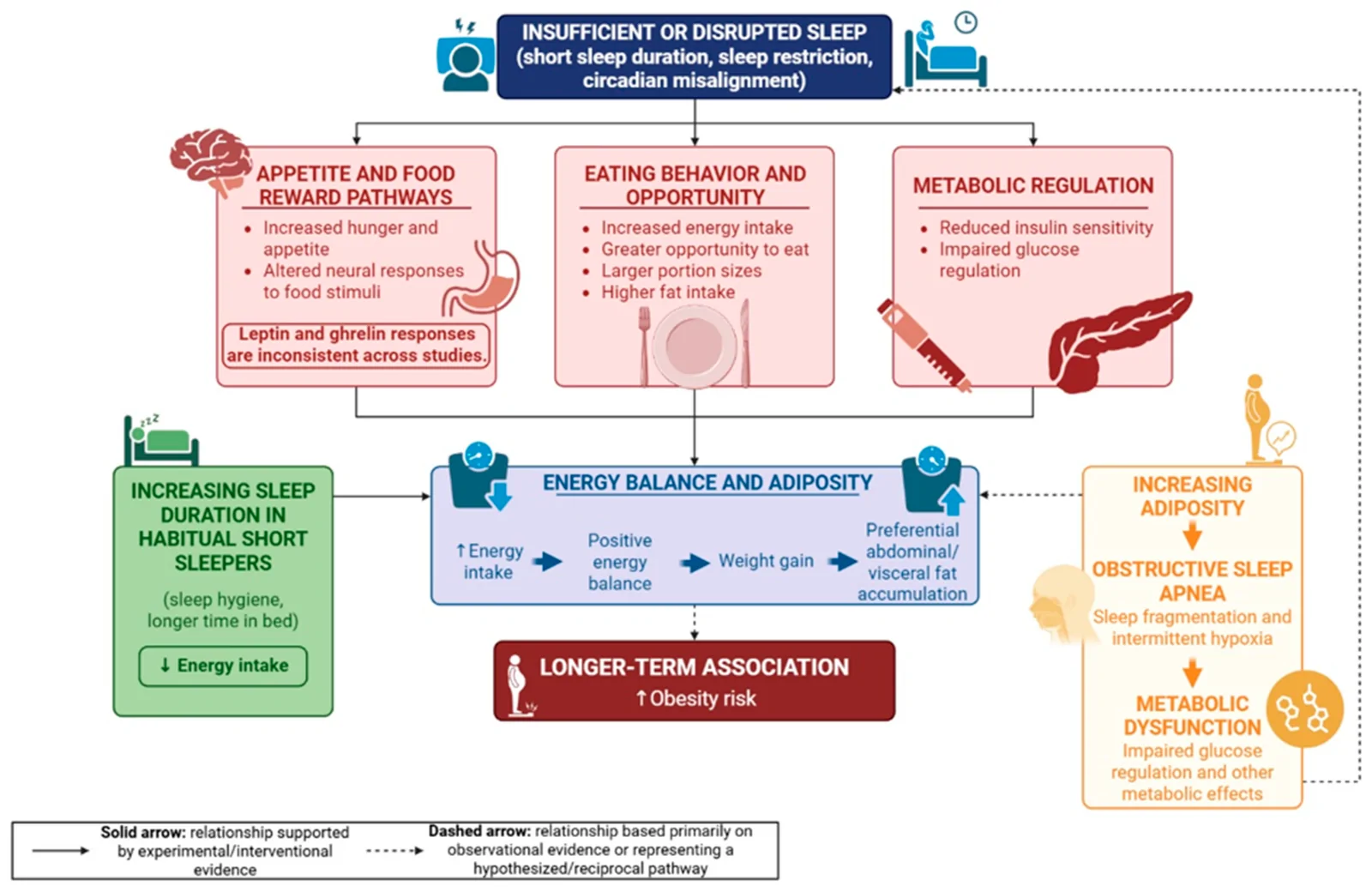
Interrelated pathways linking insufficient or disrupted sleep with appetite, eating behavior, metabolic regulation, energy balance, adiposity, obstructive sleep apnea, and longer-term obesity risk. Insufficient or disrupted sleep includes short sleep duration, experimental sleep restriction, and circadian misalignment. Experimental and interventional evidence indicates that insufficient sleep can increase hunger and appetite, alter neural responses to food stimuli, increase energy intake and eating opportunities, and impair insulin sensitivity and glucose regulation. These effects may contribute to positive energy balance, weight gain, and preferential abdominal or visceral fat accumulation. Interventions that increase habitually insufficient sleep toward an adequate duration provide evidence in the opposite direction, showing that correcting short sleep can reduce energy intake. Increasing adiposity may contribute to obstructive sleep apnea, which is characterized by sleep fragmentation and intermittent hypoxia and is associated with metabolic dysfunction, forming a potential reciprocal feedback loop with sleep and metabolic health. Solid and dashed arrows distinguish relationships supported by experimental/interventional evidence from those based primarily on observational evidence or representing hypothesized or reciprocal pathways. ↑ indicates an increase and ↓ indicates a decrease in the adjacent parameter. Internal arrows within boxes indicate conceptual sequence rather than separate evidence grading. The figure is a conceptual synthesis and does not imply that all pathways occur simultaneously or have equivalent causal support. Created in BioRender. Sorić, T. (2026) https://BioRender.com/t2dfvvn.

The available evidence does not support a single pathway linking insufficient sleep with altered dietary intake and metabolic health. Changes in appetite and food reward, greater opportunity to eat, impaired metabolic regulation, and changes in adiposity may occur in parallel rather than as a fixed sequence. Figure 2 integrates these pathways and highlights their reciprocal nature without implying that any single mechanism is necessary for the observed relationship.

### 3.3. Population-Specific Considerations in the Diet–Sleep Relationship

The relationships described in the previous sections are unlikely to operate identically across all populations. Developmental stage, aging, pregnancy, occupational circadian disruption, metabolic disease, and high physical demands can modify sleep physiology, circadian timing, nutritional requirements, or the consequences of sleep loss [22,113,114,115,116,117]. This section focuses on populations in which these modifying factors are particularly relevant to the diet–sleep relationship, including children and adolescents, pregnant women, older adults, shift workers, athletes, and individuals with cardiometabolic disease. The section is intended to highlight representative population-specific considerations rather than to provide a comprehensive review of every potentially relevant group. Population-specific evidence is nevertheless uneven, and greater physiological vulnerability does not necessarily imply stronger evidence for a specific nutritional intervention.

#### 3.3.1. Children and Adolescents

Sleep and dietary behaviors change substantially across childhood and adolescence. A systematic review of 14 studies found that longer sleep duration was generally associated with healthier dietary patterns in children and adolescents, whereas findings for sleep quality were less consistent; notably, 12 of the 14 studies were cross-sectional, with only one cohort study and one clinical trial [118].

Adolescence adds a circadian dimension because biological sleep timing shifts later while school and social schedules often continue to require early waking [114,119]. Dietary exposures used to maintain wakefulness may therefore be particularly relevant. A systematic review and meta-analysis found that adolescents with higher caffeine intake had greater odds of sleep problems (OR 1.67, 95% CI: 1.28–2.17), while alcohol consumption was associated with higher odds of insomnia (OR 1.17, 95% CI: 1.07–1.27) [120].

#### 3.3.2. Pregnancy

Sleep commonly deteriorates during pregnancy, particularly as gestation progresses. A meta-analysis found that 45.7% of pregnant women experienced poor sleep quality and that mean PSQI scores worsened significantly from the second to the third trimester [115].

The evidence linking specific dietary interventions with better sleep during pregnancy is much less developed. One clinically relevant intersection is restless legs syndrome (RLS). Meta-analytic evidence suggests that approximately one in five pregnant women experience RLS, with an overall pooled prevalence of 21% and prevalence increasing across gestation [121]. Iron deficiency has been implicated in RLS during pregnancy, making assessment of iron status clinically relevant when symptoms are present [122]. Iron supplementation should be guided by iron status and clinical indication rather than considered a general intervention for improving sleep during pregnancy.

Direct evidence that specific dietary interventions improve sleep during pregnancy remains limited. This distinction is important because correction of a clinically relevant nutritional deficiency should not be equated with the use of nutrients as sleep-promoting interventions in otherwise nutritionally replete women.

#### 3.3.3. Older Adults

Aging is accompanied by changes in sleep architecture and circadian timing, while multimorbidity, medication use, changes in appetite, and risk of nutritional inadequacy become increasingly relevant [116,123]. These factors can modify the association between diet and sleep and may partly explain why findings obtained in younger populations do not translate uniformly to later life.

In 1639 community-dwelling adults aged ≥65 years, greater adherence to the Mediterranean diet was associated with better self-reported sleep quality but not with self-reported sleep duration after adjustment for demographic, behavioral, and health-related factors. The association with sleep quality was age-dependent and was not evident in participants older than 75 years [124]. A later review focused on older adults similarly identified potentially favorable findings for Mediterranean-style diets and selected foods and nutrients, but concluded that heterogeneity in dietary exposures, sleep measures, and study designs prevented specific dietary recommendations [125].

The available evidence suggests that age may influence the relationship between dietary patterns and sleep. However, differences in study populations, dietary exposures, and sleep assessment methods limit the generalizability of current findings.

#### 3.3.4. Shift Workers

Shift work presents a distinct situation because both sleep and food intake can occur at biologically atypical times. The chrononutrition concepts discussed in Section 3.2.3 are therefore particularly relevant, but the strongest evidence concerns metabolic responses to meal timing rather than improvements in sleep itself.

Controlled laboratory studies show that the timing of food intake can modify metabolic responses to simulated night work. In a small study of healthy men, Grant et al. [126] compared participants who consumed a meal at 01:30 h during four simulated night shifts with those who avoided eating during the night. Glucose area under the curve increased from baseline after simulated night work only in the night-eating group, whereas fasting glucose and insulin did not differ significantly between conditions [126]. In a later 14-day laboratory study, Chellappa et al. [127] found that night-time eating during simulated night work shifted endogenous glucose rhythms relative to the central circadian rhythm and impaired glucose tolerance. Restricting food intake to daytime prevented both the internal circadian misalignment of glucose rhythms and the deterioration in glucose tolerance [127].

Translation into occupational practice is less straightforward. In a randomized crossover pilot trial, Leung et al. [128] studied 19 night-shift workers with abdominal obesity who habitually ate during night shifts. Participants were able to maintain a five-hour overnight fast from 01:00 to 06:00 h for four weeks, with 95% adherence, but postprandial glucose and triglyceride responses did not differ significantly from the control condition. Body weight was 0.9 kg lower after the intervention than after the control period, although this was an exploratory outcome [128].

Taken together, the experimental findings indicate that avoiding food during the biological night can attenuate some of the adverse metabolic responses to circadian disruption. Evidence from actual shift workers is less convincing, and it remains unclear whether these metabolic effects translate into improvements in sleep. Current evidence therefore supports the circadian relevance of meal timing more strongly than a specific eating schedule for improving sleep in shift workers.

#### 3.3.5. Athletes

Athletes represent a distinct context in which sleep and nutrition both contribute to recovery, while training and competition schedules, long-distance travel, and stimulant use can interfere with sleep [129,130,131]. Interest in nutritional strategies to support sleep has increased, but athlete-specific intervention evidence remains relatively limited.

Caffeine is the dietary exposure with the clearest potential to compromise sleep in this population. A systematic review of athletically trained individuals found that evening caffeine intake above approximately 2 mg/kg body mass was associated with shorter sleep duration and lower sleep efficiency, together with longer sleep latency and greater WASO [131]. This is particularly relevant because caffeine is commonly used for its ergogenic effects in sport, with performance-oriented doses often falling in the 3–6 mg/kg range [132]. The same dose that may be useful for performance can therefore become problematic when competition or training occurs later in the day.

Athlete-specific evidence for sleep-promoting foods is much smaller. Doherty et al. [133] examined two kiwifruits consumed one hour before bedtime for four weeks in elite athletes. Compared with baseline, the intervention was associated with improvements in self-reported sleep outcomes, including increased total sleep time and sleep efficiency and reductions in the number of awakenings and WASO. PSQI scores also improved [133]. The study is noteworthy because it was conducted directly in elite athletes, but it used a pre–post design without a parallel control group, so expectancy effects and changes unrelated to kiwifruit cannot be excluded.

Tart cherry products have also been tested in athletes. In a randomized controlled trial of 19 elite female field hockey players, five days of tart cherry juice supplementation improved selected actigraphy-derived sleep outcomes, including total time in bed, WASO, and movement index, while melatonin and cortisol concentrations were unchanged [134]. Although this provides athlete-specific intervention evidence, the small sample and short intervention period make it difficult to draw broader conclusions regarding habitual sleep improvement.

The limited size of this literature is apparent in a recent scoping review of nutritional strategies intended to promote sleep in elite athletes. Only 12 studies met the inclusion criteria, and the median intervention group size was 19 participants. Kiwifruit, tart cherry juice, and higher dairy intake in female athletes showed the most promising findings, but the authors emphasized the small samples and lack of replication across sports and populations [135].

Evidence concerning nutritional interventions to improve sleep in athletes remains limited, and the available findings require confirmation in larger controlled studies.

#### 3.3.6. Cardiometabolic Disease

The reciprocal relationship between sleep, dietary intake, and metabolic regulation described in Section 3.2.5 is especially relevant in obesity, insulin resistance, and type 2 diabetes. In people with type 2 diabetes, both sleep duration and sleep quality have been associated with glycemic control. In a systematic review and meta-analysis, short sleep was associated with a 0.23-percentage-point higher hemoglobin A1c (HbA1c) and long sleep with a 0.13-percentage-point higher HbA1c compared with the reference sleep duration of 6–8 h/night, while poor sleep quality was associated with a 0.35-percentage-point higher HbA1c [136]. These relationships are difficult to disentangle from adiposity, OSA, and other lifestyle and treatment-related factors that influence both sleep and metabolic outcomes [117,137].

Intervention evidence is less consistent. In adults with type 2 diabetes and insomnia, cognitive behavioral therapy for insomnia improved insomnia symptoms and mood, while the improvement in glycemic control did not reach statistical significance [138]. An earlier systematic review similarly found that sleep interventions generally improved sleep, but effects on glucose metabolism were inconclusive [139]. More recent meta-analytic evidence is somewhat more encouraging. Mostafa et al. [140] found that cognitive behavioral therapy for insomnia and/or sleep-hygiene interventions reduced HbA1c by 0.27 percentage points overall and by 0.43 percentage points among people with type 2 diabetes, whereas fasting glucose and glycemic outcomes following sleep-extension interventions did not change significantly. Differences in intervention type, baseline sleep disturbance, duration, and participant characteristics may partly explain the variation between studies.

Evidence for whether dietary modification can improve sleep in this population is also limited. In the MEDGICarb randomized trial, adults with overweight or obesity and cardiometabolic risk consumed Mediterranean-style diets differing in glycemic index. After 12 weeks, the low- and high-glycemic-index groups did not differ significantly in self-reported sleep quality or daytime sleepiness, assessed using the PSQI and Epworth Sleepiness Scale, respectively [141]. These findings suggest that improvements in metabolic health following dietary modification do not necessarily translate into better sleep.

Although sleep disturbances and cardiometabolic dysfunction are closely associated, studies examining dietary modification, sleep interventions, and metabolic outcomes address different research questions. Evidence that dietary modification independently improves sleep in individuals with cardiometabolic disease remains limited.

## 4. Discussion

### 4.1. Translation of Current Evidence into Practice

Translating findings on nutrition and sleep into practice requires careful consideration of the strength and type of evidence available. Controlled studies provide relatively direct evidence for some exposures, particularly caffeine and alcohol, while findings for dietary patterns, individual foods, supplements, and chrononutrition rely more heavily on observational studies or small interventions.

For caffeine and alcohol, the practical implications are relatively clear. Caffeine intake should be considered in relation to dose, timing of intended sleep, habitual use, and individual sensitivity, particularly when it is used to support alertness or performance [53,54,95,131,132]. Alcohol should not be used as a strategy to facilitate sleep, as its sedative effects do not reflect an improvement in sleep overall [55,56].

Mediterranean-style and other higher-quality dietary patterns can be recommended for their established overall health benefits, with any improvement in sleep regarded as a possible additional benefit rather than a therapeutic effect [4,7,42]. Specific foods such as kiwifruit, tart cherry products, dairy products, and fatty fish should likewise be considered within an overall nutritious diet rather than prescribed as sleep-promoting interventions [44,45,47,48,83].

For magnesium and vitamin D, the available evidence does not support routine supplementation solely to improve sleep in nutritionally replete individuals. When deficiency or another clinical indication is present, assessment and treatment should follow established clinical practice rather than be based on sleep symptoms alone [40,68].

Meal timing may also be relevant, particularly when eating occurs at an adverse circadian phase, but current evidence does not support a universal meal time or eating window for improving sleep. Similarly, time-restricted eating should not currently be prescribed primarily as a sleep intervention [11,52,88,89,92,127]. Table 3 summarizes what the current evidence can and cannot support in practice.

Overall, the evidence is stronger for identifying and modifying dietary exposures that can impair sleep than for recommending foods, supplements, or specific eating schedules to improve it. Caffeine and alcohol provide the clearest examples, while evidence for individual foods, supplements, and meal-timing strategies remains less consistent. Nutritional approaches should complement, rather than replace, appropriate evaluation and management of clinically relevant sleep disorders.

### 4.2. Critical Appraisal and Limitations of the Evidence

Much of the evidence linking dietary patterns, diet quality, and habitual meal timing with sleep comes from observational studies, making residual confounding a persistent concern. Artificial light exposure, occupational and psychological stress, physical activity, and socioeconomic circumstances may influence both eating behaviors and sleep. For example, exposure to artificial light at night has been associated with shorter sleep duration [142], while socioeconomic circumstances can influence food choices, working conditions, and the sleep environment [143]. Physical activity is another relevant factor because it is associated with sleep and often accompanies healthier dietary behaviors. These factors frequently coexist and are not consistently measured or adequately controlled for in observational research. Consequently, associations attributed to dietary exposures may partly reflect differences in lifestyle, environmental conditions, or socioeconomic circumstances.

Insufficient sleep can alter appetite, food preferences, and subsequent energy intake, as demonstrated in experimental studies discussed in Section 3.2.5. A randomized crossover study by Dickinson et al. [144] also reported changes in physical activity following experimental sleep restriction, although its findings concerning dietary behavior were less consistent. Dietary assessment methods introduce further uncertainty. Both 24-h dietary recalls and food frequency questionnaires are susceptible to reporting errors, particularly when participants have difficulty recalling food consumption or estimating portion sizes [145]. Sleep deprivation can impair attention and aspects of cognitive performance [146], potentially affecting the accuracy of retrospective dietary reporting. However, direct evidence demonstrating that sleep deprivation systematically alters dietary recall accuracy remains limited. The possibility of sleep-related reporting bias should be considered without assuming that its magnitude or direction has been established.

Genetic factors may influence caffeine metabolism and sleep responses, but their clinical relevance remains uncertain. Polymorphisms in *CYP1A2*, including rs762551, have been investigated in relation to caffeine metabolism [94,95], while variation in *ADORA2A* has been associated with caffeine-related sleep responses [147]. Circadian clock genes, including *CLOCK* and *PER2*, have also been examined in relation to chronotype, sleep, and temporal eating patterns. Associations involving individual variants are inconsistent, and evidence that these variants predict responses to meal-timing interventions is insufficient [148]. Accordingly, the clinical relevance of genetic variation for personalized chrononutrition remains to be established.

Interpretation is further complicated by differences in study populations, intervention protocols, baseline nutritional status, and sleep assessment methods. Many intervention studies also involve relatively small samples, short intervention periods, or sleep assessments conducted as secondary outcomes. These limitations restrict the strength of conclusions that can be drawn, particularly regarding the clinical effectiveness of individual foods, supplements, and meal-timing interventions.

### 4.3. Knowledge Gaps and Priorities for Future Research

Prospective studies with repeated assessments of dietary intake and sleep are needed to clarify the direction of observed associations. These studies should account more consistently for environmental, occupational, behavioral, and socioeconomic factors, including artificial light exposure, work schedules, psychological stress, and physical activity. Combining repeated dietary assessments with prospective food records and objective sleep measurements, where appropriate, could help address some of the limitations associated with retrospective dietary reporting. Chrononutrition research requires greater methodological consistency in the definition and assessment of temporal eating patterns. Meal timing, eating-window duration, eating regularity, and eating frequency represent distinct exposures, but their measurement varies considerably across studies [11,149]. Recent methodological recommendations have emphasized standardized terminology and time-stamped dietary assessment [150]. Greater consideration of individual circadian timing is also necessary. For example, an evening meal consumed at the same clock time may occur at different biological times in individuals with early and late chronotypes. Future studies should therefore report meal timing in relation to habitual sleep schedules and, where feasible, objectively assessed circadian phase [11,151]. Objective circadian markers such as dim-light melatonin onset (DLMO) may be particularly informative in mechanistic or tightly controlled studies [152].

Intervention studies should distinguish between the effects of nutritional interventions in individuals with inadequate nutrient status and those who are nutritionally replete. Baseline nutritional status is particularly relevant when evaluating magnesium, vitamin D, and other nutrients for which deficiency may influence the response to supplementation. Studies intended to improve sleep should also specify sleep as a primary outcome rather than assess it only as a secondary endpoint. This is particularly important in time-restricted eating research, where many interventions have primarily targeted body weight or cardiometabolic outcomes [88].

Individual variability deserves greater attention, particularly in relation to chronotype, habitual sleep patterns, metabolic status, and genetic factors. However, the potential contribution of genetic variation should be examined in adequately powered studies designed to test interactions between genotype and nutritional interventions. Associations between circadian gene variants and eating behaviors alone are insufficient to establish genotype-specific recommendations. Future trials should consider whether statistically significant changes in sleep outcomes translate into clinically meaningful improvements. Larger samples, longer intervention periods, appropriate objective sleep measurements, preregistered outcomes, and replication across different populations would help establish the clinical relevance and reproducibility of reported findings. These improvements are particularly important before recommending individual foods, supplements, or specific eating schedules as interventions for sleep problems.

## 5. Conclusions

### 5.1. Key Conclusions

The evidence reviewed here supports a clear distinction between dietary exposures with relatively well-established effects on sleep and those for which the evidence remains preliminary.

More consistently supported findings:

- Caffeine can disrupt sleep in a dose- and timing-dependent manner, although individual sensitivity varies and a universal cut-off time cannot currently be defined.
- Alcohol can alter sleep architecture, particularly REM sleep, and should not be regarded as a sleep-promoting strategy.
- Higher-quality dietary patterns, particularly Mediterranean-style diets, are consistently associated with more favorable sleep characteristics, although the predominantly observational evidence does not establish a causal sleep benefit.

Findings that remain preliminary or inconsistent:

- Evidence that individual foods or nutrients, including kiwifruit, tart cherry products, dairy products, magnesium, and vitamin D, improve sleep is limited and does not support their use as established sleep interventions.
- Time-restricted eating and other meal-timing interventions have shown inconsistent effects on sleep and cannot currently be recommended specifically for this purpose.
- Genetic differences may contribute to variability in responses to caffeine and meal timing, but current evidence is insufficient to support genotype-based chrononutrition recommendations.

Sleep can also influence dietary intake, appetite, energy balance, and metabolic health, emphasizing the bidirectional nature of the relationship. Nutrition should therefore be considered as one component of a broader approach to sleep health rather than as a stand-alone treatment for sleep disorders.

### 5.2. Limitations of This Review

This review has several limitations. As a narrative rather than systematic or meta-analytic review, it did not use a single predefined search strategy across all topics, calculate pooled effect estimates, or formally assess risk of bias or certainty of evidence for individual studies. The review was limited to peer-reviewed publications available in English, so relevant evidence published in other languages may have been missed. In addition, some of the literature on individual foods and supplements is based on small studies, which may be particularly susceptible to publication bias and selective reporting of favorable findings. Finally, the broad scope of the review required synthesis across heterogeneous exposures, populations, and sleep outcomes, limiting the depth with which some individual topics could be examined.

### 5.3. Priorities for Future Study Design

Future studies should prioritize methodologically rigorous, adequately powered studies using standardized dietary and sleep assessments, clearly prespecified sleep outcomes, and appropriate characterization of baseline nutritional and sleep status. Greater consistency in study design and replication across populations will be important for determining whether observed effects are reproducible and clinically meaningful.

## Figures and Tables

**Table 1:** Characteristics and principal findings of representative studies and evidence syntheses examining the relationship between nutrition and sleep.

Exposure/Intervention	Study Design	Participants or Included Studies	Assessment Method	Principal Reported Result
Tryptophan supplementation [38]	Systematic review with meta-analysis and meta-regression	18 articles identified; four contributed to the quantitative meta-analysis	WASO; assessment methods varied	Tryptophan supplementation was associated with shorter WASO (SMD = −1.08, 95% CI −1.89 to −0.28). Participants receiving ≥1 g/day had shorter WASO than those receiving <1 g/day (28.91 vs. 56.55 min; *p* = 0.001).
Omega-3 fatty acids [39]	Systematic review and meta-analysis of randomized controlled trials	Eight studies overall; five contributed six comparisons to the sleep-efficiency analysis	Objective and subjective sleep measurements	Sleep efficiency improved by 1.88 percentage points (95% CI 1.00–2.77 percentage points); no statistically significant effect on sleep latency or duration.
Magnesium supplementation [40]	Systematic review and meta-analysis of randomized controlled trials	Three trials; 151 older adults with insomnia	Sleep-onset latency and total sleep time; methods varied	Sleep-onset latency decreased by 17.36 min relative to placebo (95% CI −27.27 to −7.44; *p* = 0.0006). Total sleep time was not significantly different.
Vitamin D status [41]	Systematic review and meta-analysis of observational studies	Nine studies; 9397 participants	Study-specific sleep assessments	Vitamin D deficiency was associated with greater odds of sleep disorders (OR 1.50, 95% CI 1.31–1.72) and poor sleep quality (OR 1.59, 95% CI 1.23–2.05).
Overall dietary patterns and diet quality [4]	Systematic review	29 studies	Sleep-quality measures varied	Healthier foods and dietary patterns were generally associated with better sleep quality. No single pooled effect was reported across all exposures.
Mediterranean diet adherence [42]	Systematic review and meta-analysis of observational studies	10 studies (9 publications); 13,511 participants	Insomnia symptoms	Higher adherence was associated with lower odds of insomnia symptoms (OR 0.86, 95% CI 0.79–0.93; *p* < 0.001).
Ultra-processed food consumption [43]	Systematic review and meta-analysis	Seven studies in the pooled insomnia analysis; 107,194 participants	Insomnia outcomes	Higher consumption was associated with insomnia (OR 1.53, 95% CI 1.20–1.95). The subgroup association was observed in adolescents but not adults.
Kiwifruit [44]	Uncontrolled before-and-after intervention	24 adults	Chinese PSQI, sleep diaries, and actigraphy	After four weeks, PSQI scores decreased by 42.4%, sleep-onset latency by 35.4%, and WASO by 28.9%; total sleep time increased by 13.4% and sleep efficiency by 5.41%.
Tart cherry juice [45]	Randomized, double-blind, placebo-controlled crossover pilot study	11 randomized; eight analyzed	Polysomnography and questionnaires	Polysomnographic total sleep time increased by 84 min versus placebo (*p* = 0.0182); polysomnographic sleep efficiency did not improve significantly.
Montmorency tart cherry powder [46]	Randomized placebo-controlled crossover study	34 participants completed the study	Zmachine single-channel EEG, Fitbit, PSQI, and Insomnia Severity Index	No significant between-treatment differences in objective sleep outcomes, PSQI (*p* = 0.51), or Insomnia Severity Index (*p* = 0.92).
Milk and dairy products [47]	Systematic review and meta-analysis	Nine trials in qualitative synthesis; four in the PSQI meta-analysis	PSQI	Milk and dairy consumption was associated with lower PSQI scores (pooled mean difference −0.43, 95% CI −0.67 to −0.20; *p* = 0.0003).
Atlantic salmon [48]	Randomized dietary intervention	95 male forensic inpatients randomized; outcome-specific sample sizes varied	Actigraphy and daily self-report diary	Sleep latency increased in the control group but not the fish group (group-by-time interaction *p* = 0.05); self-reported daily functioning differed at follow-up.
Meal timing [49]	Cross-sectional analysis	7023 US adults	PSQI and two 24-h dietary recalls	Latest versus earliest tertile adjusted PSQI differences: first meal +0.545 (95% CI 0.226–0.864), midpoint +0.586 (0.277–0.896), and last meal +0.385 (0.090–0.680).
Dinner timing [50]	Randomized crossover study; post hoc sleep analysis	20 healthy adults	Polysomnography and EEG spectral analysis	Conventional sleep-stage distribution did not differ significantly. Initial delta power increased by 2.5%, while combined alpha/beta power decreased by 2.7% with late dinner (*p* < 0.0001).
Early time-restricted eating [51]	Post hoc sleep analysis of randomized controlled feeding trial	38 participants with usable actigraphy data	Wrist actigraphy	Total sleep time increased by 55 min (*p* = 0.03), and sleep midpoint shifted 44 min earlier (*p* = 0.01), compared with usual eating.
Self-selected 10-h TRE [52]	Randomized controlled trial	30 young adults	Actigraphy and PSQI	Earlier sleep onset and later waking (both *p* < 0.001), with fewer movements during sleep (*p* < 0.025); no significant between-group difference in sleep duration.
Caffeine dose and timing [53]	Randomized, double-blind, placebo-controlled crossover trial	23 healthy men	Partial polysomnography and sleep diaries	At 400 mg, caffeine altered sleep outcomes even when consumed 12 h before bedtime. Subjective sleep quality was 34.02% lower when consumed 4 h before bedtime (*p* = 0.006).
Caffeine, pooled experimental evidence [54]	Meta-analysis of controlled crossover trials	22 trials; 956 participants	Polysomnography	Total sleep time decreased by 34.67 min, sleep efficiency by 4.74 percentage points, and sleep-onset latency increased by 8.35 min.
Alcohol and REM sleep [55]	Systematic review and meta-analysis	27 experimental studies	Sleep architecture and REM outcomes	Delayed REM onset and shorter REM duration were reported from doses ≤ 0.50 g/kg, with greater disruption at higher doses.
Repeated pre-sleep alcohol [56]	Crossover laboratory study	30 adults	Polysomnography	Alcohol increased slow-wave sleep accumulation and delayed or reduced early-night REM accumulation across consecutive nights.
Alcohol and incident sleep disorders [57]	Systematic review and meta-analysis	11 cohort studies	Incident sleep disorders	General drinking: OR 1.37 (95% CI 1.22–1.54); heavy drinking subgroup: OR 1.22 (95% CI 0.94–1.60).
Energy-drink consumption [58]	Cross-sectional national student survey	53,266 Norwegian students	Self-reported consumption and sleep	Daily consumption was associated with short sleep, with RR 2.07 (95% CI 1.77–2.42) in men and 1.87 (1.64–2.14) in women.
Experimental sleep restriction [59]	Systematic review and meta-analysis	41 randomized trials	Hunger, energy intake, and body weight	Hunger increased by 13.4 mm on a 100-mm visual analogue scale (*p* < 0.001), energy intake by 252.8 kcal/day (*p* = 0.011), and body weight by 0.34 kg following partial sleep restriction (*p* = 0.003).
Increasing sleep duration in habitual short sleepers [60]	Randomized controlled trial	80 adults with overweight who habitually slept <6.5 h/night	Actigraphy and objectively estimated energy intake	Sleep increased by 1.2 h/night (95% CI 1.0–1.4), while energy intake decreased by 270 kcal/day (95% CI −393 to −147; *p* < 0.001).

Abbreviations: CI, confidence interval; SMD, standardized mean difference; EEG, electroencephalography; OR, odds ratio; PSQI, Pittsburgh Sleep Quality Index; REM, rapid eye movement; RR, risk ratio; TRE, time-restricted eating; WASO, wake after sleep onset. Note: The table presents representative publications, including systematic reviews and meta-analyses; the number of studies in a review is not a participant sample size. Numeric estimates reflect the cited publications and should not be compared directly across exposure types or outcome instruments. Where a single participant total or pooled effect was not applicable or reliably provided for the outcome, the study count or descriptive finding is shown.

**Table 2:** Main chrononutrition dimensions relevant to sleep and their assessment in human studies.

Chrononutrition Dimension	What Is Being Assessed	Examples of Operational Measures	Main Methodological Consideration
Meal timing	Timing of meals or eating occasions across the day	Timing of first and last eating occasion; breakfast, lunch or dinner timing; eating midpoint	Definitions and reference points used to characterize early or late eating vary across studies
Distribution of intake across the day	Temporal distribution of energy or nutrient intake	Energy or nutrient intake across predefined periods of the day	Time periods and methods used to quantify intake distribution are not standardized
Eating-window duration	Duration of the daily period in which energy is consumed	Interval between first and last caloric intake; daily eating-window duration	Eating-window duration does not by itself describe when the window occurs within the day
Eating regularity	Consistency of eating patterns across days	Variability in meal timing; meal skipping; day-to-day differences	Regularity is conceptualized and quantified differently across studies
Eating frequency	Frequency of eating occasions	Number of meals, snacks or total eating occasions per day	Definitions of meals, snacks and eating occasions differ across studies

Note: Chrononutrition dimensions and methodological considerations were informed by Saidi et al. [11] and O’Connor et al. [84].

**Table 3:** What current nutrition–sleep evidence can and cannot support in practice.

Exposure or Strategy	Evidence Summary	What the Evidence Does Not Establish	Practical Interpretation
Caffeine	Sleep-disrupting effects are supported by controlled evidence and depend on dose and timing [53,54]	A single cut-off time appropriate for all individuals	Consider dose and timing relative to intended sleep, together with habitual intake and individual sensitivity
Alcohol	Controlled evidence supports disruption of sleep architecture, particularly REM sleep [55,56]	That its sedative effect translates into better overall sleep	Alcohol should not be used specifically to facilitate sleep
Mediterranean-style/higher-quality dietary patterns	Observational evidence consistently favors an association with better sleep characteristics [4,7,42]	That dietary pattern modification alone treats insomnia or reliably improves sleep	Recommend for established overall health benefits; better sleep may be an additional benefit
Specific foods	Food-specific intervention evidence is promising but limited and heterogeneous [44,45,47,48,83]	Established therapeutic or generalizable sleep effects of individual foods	Consider within an overall nutritious diet rather than as specific sleep treatments
Magnesium and vitamin D	Intervention evidence suggests possible benefits for selected sleep outcomes, but remains limited [40,68]	Routine sleep benefits from supplementation in nutritionally replete individuals	Correct deficiency or inadequacy when clinically indicated rather than supplement solely for sleep
Meal timing/chrononutrition	Circadian relevance is supported, but evidence directly linking meal-timing interventions to better sleep remains limited [11,49,85,127]	A universal optimal meal time or schedule for improving sleep	Consider meal timing in relation to habitual sleep and circadian schedule
Time-restricted eating	Controlled evidence shows no consistent effect on sleep, despite favorable findings in selected studies [52,88,92]	Consistent improvement in sleep quality, duration, or insomnia	Current evidence does not support prescribing TRE primarily as a sleep intervention
Increasing sleep duration in habitual short sleepers	Experimental evidence indicates that increasing sleep duration can reduce energy intake in habitual short sleepers [60]	Benefits of extending sleep beyond adequate duration or reliable long-term weight loss from sleep-duration intervention alone	The potential benefits of increasing sleep duration are most relevant to individuals with habitually insufficient sleep

Abbreviations: REM, rapid eye movement; TRE, time-restricted eating.

## Data Availability

No new data were created or analyzed in this study. Data sharing is not applicable to this article.

## Supplementary Materials

The following supporting information can be downloaded at: https://www.mdpi.com/article/10.3390/clockssleep8040067/s1, Table S1: Descriptive overview of how the SANRA domains are addressed in the review.

## Abbreviations

The following abbreviations are used in this manuscript:

CI	confidence interval
CYP1A2	cytochrome P450 1A2
DASH	Dietary Approaches to Stop Hypertension
DLMO	dim-light melatonin onset
EEG	electroencephalography
HbA1c	hemoglobin A1c
MCTQ	Munich Chronotype Questionnaire
MeSH	Medical Subject Headings
MEQ	Morningness–Eveningness Questionnaire
MIND	Mediterranean–DASH Intervention for Neurodegenerative Delay
NREM	non-rapid eye movement
OR	odds ratio
OSA	obstructive sleep apnea
PRISMA	Preferred Reporting Items for Systematic Reviews and Meta-Analyses
PSQI	Pittsburgh Sleep Quality Index
REM	rapid eye movement
RLS	restless legs syndrome
RR	risk ratio
SANRA	Scale for the Assessment of Narrative Review Articles
SMD	standardized mean difference
TRE	time-restricted eating
WASO	wake after sleep onset
