# Supplementary material for: Chrononutrition, Dietary Patterns, and Sleep: A Narrative Review of Current Evidence and Practical Implications
Source: Clocks Sleep. 2026 Oct 15;8(4):67. doi: 10.3390/clockssleep8040067 (PMC13692510; doi:10.3390/clockssleep8040067)
Supplement: Supplementary file 1 [file clockssleep-08-00067-s001.zip › clockssleep-4568591-supplementary.pdf]

Table S1. Descriptive overview of how the SANRA domains are addressed in the review.

SANRA domain	How it is addressed in this review
Justification of the review's importance	Sections 1.1 and 1.2 explain why nutrition, sleep, and circadian health should be considered together and identify the need for a review that integrates dietary composition, meal timing, sleep-disrupting dietary exposures, and the effects of insufficient sleep on dietary intake and metabolic health.
Statement of aims	Section 1.3 states the primary aim, defines the scope of the review, and presents three focused questions addressing dietary patterns and individual nutrients and foods, temporal eating patterns and circadian factors, and population-specific considerations. The broader scope also includes caffeine and alcohol and the consequences of insufficient sleep for dietary intake and metabolic health.
Description of the literature search	Section 2.1 reports the databases searched, the search period, representative search combinations, and the use of additional topic-specific terms. Sections 2.2 and 2.3 describe inclusion and exclusion criteria, literature selection, and narrative synthesis.
Referencing	Mechanistic statements, observational associations, intervention findings, and practical conclusions are linked to the corresponding primary studies, systematic reviews, or meta-analyses. Where several publications addressed the same question, greater weight was given to higher-level and prospective evidence when available.
Scientific reasoning	Throughout the review, observational findings are distinguished from experimental and interventional evidence. Interpretation takes into account confounding, reverse causation, temporality, heterogeneity in study design, and differences in dietary and sleep assessment. These limitations are discussed explicitly in Sections 3 and 4.
Appropriate presentation of relevant outcome data	Quantitative findings are reported where available, including sample sizes, effect estimates, confidence intervals, p-values, and absolute changes. The review also distinguishes subjective from objective sleep measures and avoids presenting findings from different study designs or outcome measures as directly comparable.

Note: SANRA, Scale for the Assessment of Narrative Review Articles. SANRA was used as a framework to evaluate the reporting and methodological transparency of the narrative review and not as a risk-of-bias assessment of the individual studies included in the synthesis.
